# Concise Review: Polarity in Stem Cells, Disease, and Aging

**DOI:** 10.1002/stem.481

**Published:** 2010-07-16

**Authors:** Maria Carolina Florian, Hartmut Geiger

**Affiliations:** a Department of Dermatology and Allergic Diseases, University of UlmUlm, Germany; b Division of Experimental Hematology and Cancer Biology, Cincinnati Children's Hospital Medical CenterCincinnati, Ohio, USA

**Keywords:** Stem cell, Polarity, Cdc42, Aging, Quiescence, Asymmetric division

## Abstract

Adult somatic stem cells are central to homeostasis in tissues that present with a high cellular turnover like the skin, intestine, and the hematopoietic system. It is thought that polarity is particularly important with respect to fate decisions on stem cell division (symmetric or asymmetric) as well as for the maintenance of stem cell adhesion and quiescence (interaction with the niche). Consequently the failure to establish or regulate stem cell polarity might result in disease or tissue attrition. Members of the family of small RhoGTPases are known to exert an important role in regulating cell polarity. We summarize and discuss here recent views on the role of cell polarity in somatic stem cell function, aging, and disease, concluding that targeting cell polarity might be a novel approach to ameliorate or even revert aberrant somatic stem cell function. Stem Cells 2010; 28:1623–1629.

## INTRODUCTION

Somatic stem cells are central to tissue homeostasis. In this review, we summarize the data available on somatic stem cell polarity and discuss its importance for stem cell function. We first describe structures of polarity and depict pathways on how a polar distribution of proteins is achieved. Mechanistically, we focus primarily on the role of the small Rho GTPase Cdc42 in establishing polarity [1–3]. Next, we outline in more detail published data on polarity in stem cells and conclude with a discussion on the role of polarity with respect to symmetric versus asymmetric stem cell division, stem cell aging, and cancer.

## CELL POLARITY: CONCEPTS, ORGANELLES, AND STRUCTURES

A cell can be defined as polarized when organelles, proteins, mRNAs, and/or microRNAs inside it are distributed and maintained in a nonsymmetrical organization. Cell polarization can occur in response to extracellular stimuli that induce a redistribution of cellular components to fulfill a functional need during adhesion, migration, or cell division. For example, a migrating cell is characterized by a protruding front and a retracting rear, while the cell polarity axis is oriented in a direction defined by the external chemoattractant, with adhesion being the most frequent initiating event for defining morphological subdomains, at least in eukaryotic cells. The orientation of the polar axis in such a cell can be determined by the shape of the cell, the direction of cell protrusions, the orientation of microtubule and actin networks, and the position of the centrosome/Golgi complex relative to that of the nucleus. Multiple distinct structural components with a polar distribution in a eukaryotic cell have been described which we will review briefly in the following sections.

### Cell Polarity and the Plasma Membrane

Several studies have shown that plasma membrane domains with specialized lipid composition are distributed asymmetrically in polarized cells. These domains are commonly enriched in cholesterol and are described as lipid rafts. Different proteins (membrane receptors, adaptor proteins involved in signal transduction, effectors such as small GTPases and heterotrimeric G proteins, or activators and inhibitors of effector proteins) can redistribute in association with their respective raft to accomplish a specific cell function. For example, in migrating lymphocytes, membrane-anchored cell surface receptors such as CXCR4, CCR5, CD44, and Inter-cellular adhesion molecule (ICAM), among others, associate with lipid rafts, together with their respective signal transducing molecules (mainly heterotrimeric G-proteins). This polar distribution of receptors and downstream signaling proteins is critical for achieving highly compartimentalized signal transduction to regulate lymphocyte function during extravasation or crawling [4,5]. In addition, several scaffold proteins that can recruit subdomain-specific membrane-bound receptors and signaling transduction proteins are important for polarity at the membrane. Reggie or flotillin is an example of such scaffolding proteins for the assembly of multiprotein signaling complexes in microdomains. They have been implicated in regulating several critical cell signaling pathways [6] and are able to form polar clusters at the cytoplasmatic side of the plasma membrane by homo- and hetero-oligomerization.

### Cell Polarity, Cytoskeleton, and Centrosome

Actin microfilaments and microtubules are polarized polymers, which can establish polarity inside the cell. For example, Siegrist and Doe described that cell-intrinsic position-signals from the microtubule cytoskeleton are sufficient to induce and/or maintain cell polarity, ranging from yeast cells to human fibroblasts [7].

The centrosome is the primary microtubule-organizing center (MTOC) and functions as the cell intrinsic chiral center. It usually contains a pair of centrioles in mammalian cells. The two centrioles are oriented at right angles and consist of triplet microtubules. Because microtubules assemble from the MTOC, polarity becomes fixed in a specific orientation with the (−) ends of microtubules closest to the MTOC. Even in the absence of outside spatial cues, the centrosome can serve as a template for directing polarity. It has been thus associated with establishing planar polarity during asymmetric cell division [8]. In addition, recent data indicate that centrosome activity and/or its positioning is regulated by cell-adhesion proteins, serving as a link to regulate organelle position in response to external cues [9–11]. Furthermore, the centrosome has recently been identified as a proteolytic center of the cell, as in many cell types proteasomes are concentrated at the centrosome [12]. Interestingly, in some cell types proteins targeted for degradation are inherited preferentially by one daughter centrosome during cell division, resulting in transmission of a distinct type of asymmetry, and thus of polarity, during mitosis. Although phospho-Smad1 targeted for proteasomal degradation is asymmetrically distributed during mitosis in human embryonic stem cells (hESCs), total Smad1 is uniform. The asymmetric segregation of phopsho-Smad1 takes place during self-renewing divisions of cultured hESCs and seems to be explained by the asymmetric inheritance of pericentrosomal proteins at the time the centrioles separate during G_2_/M transition [12].

### Cell Polarity and the Golgi Apparatus

Endomembranes and particularly the Golgi apparatus play an important role in conferring cell polarity, mostly via regulating the centrosome. The Golgi apparatus functions in localizing specific proteins for centrosome-related processes that ultimately regulate centrosome organization and spindle formation during mitosis [13]. Moreover, it is also known that centrosome/Golgi complex is directly involved in regulating polarity during cell migration as on stimulation it can reorient to the leading edge of a migrating cell [14,15].

Which events initiate the rearrangement of cellular components/structures in a polar fashion? In an inductive model of establishment of polarity, a cell orients proteins and organelles in a polar manner in response to a chemoattractant or in response to contacting other surrounding cells or the extracellular matrix. Alternatively, in an intrinsic model, polarity results from intrinsic (and mostly unknown) mechanisms, with the consequence that external reactions (adhesion to other cells or the extracellular matrix, directional migration) are secondary to the previously established intrinsic initiating event. Most likely both of these modes of initiation are found in nature, and the specific contribution of both of these mechanisms might depend on the type of cell as well as the functional needs of the cell [16].

## MOLECULAR MECHANISMS OF CELL POLARITY: THE ROLE OF THE SMALL RHOGTPASE Cdc42

By assembling multiprotein complexes in distinct parts of the cell polarity proteins induce downstream signaling events to trigger the establishment of cellular polarity. At least three different major polarity protein complexes [the partition protein (PAR), Crumbs, and Scribble], initially characterized in *C. elegans* and *D. melanogaster*, have been subsequently identified also in eukaryotic cells. How these polarity complexes influence cell polarity in general has been recently discussed elsewhere [17,18]. We thus focus here only briefly on the role of the small RhoGTPase Cdc42, a protein either associated with polarity complexes like the PAR complex and/or part of both upstream and downstream signaling events of polarity complexes.

Cdc42 belongs to one of the subfamilies of small RhoGTPases and its role in establishing polarity primarily through interaction with the PAR polarity complex has been reported for cells ranging from yeast to mammals [19,20]. Cdc42 seems to be absolutely essential in mammals as the *Cdc42*-knockout phenotype in mice is embryonic lethal with death before embryonic day 7.5 [21]. Moreover, Cdc42 is required for the establishment of the apical-basal axis in epithelial cells and in differentiating neurons [1–3], and interacts with proteins that regulate endocytosis and mediate vesicular trafficking between the endoplasmic reticulum and the Golgi [22,23]. It controls epithelial tissue morphogenesis by regulating spindle orientation during cell division [24] and modulates cell adhesion and polarity during embryonic morphogenesis by regulating the trafficking of key cell junction proteins [25,26].

The analysis of distinct tissues from *Cdc42*-knockout mouse embryos has demonstrated that regulation of polarity by Cdc42 in vivo is important in fate determination of several different cell types, such as neural progenitor cells at the apical ventricular zone, epidermal keratinocytes, and bone marrow progenitor cells [1,20,27–29]. Finally, misregulation of Cdc42-driven cell polarity pathways is also linked to cellular transformation. For example, it has been shown that aberrant activation of Cdc42 results in tumorigenesis and tumor progression besides initiation of cardiovascular diseases, diabetes, and neuronal degenerative diseases [30].

## POLARITY AND STEM CELLS: DIVISION, QUIESCENCE, AND MIGRATION

**Figure 1:**
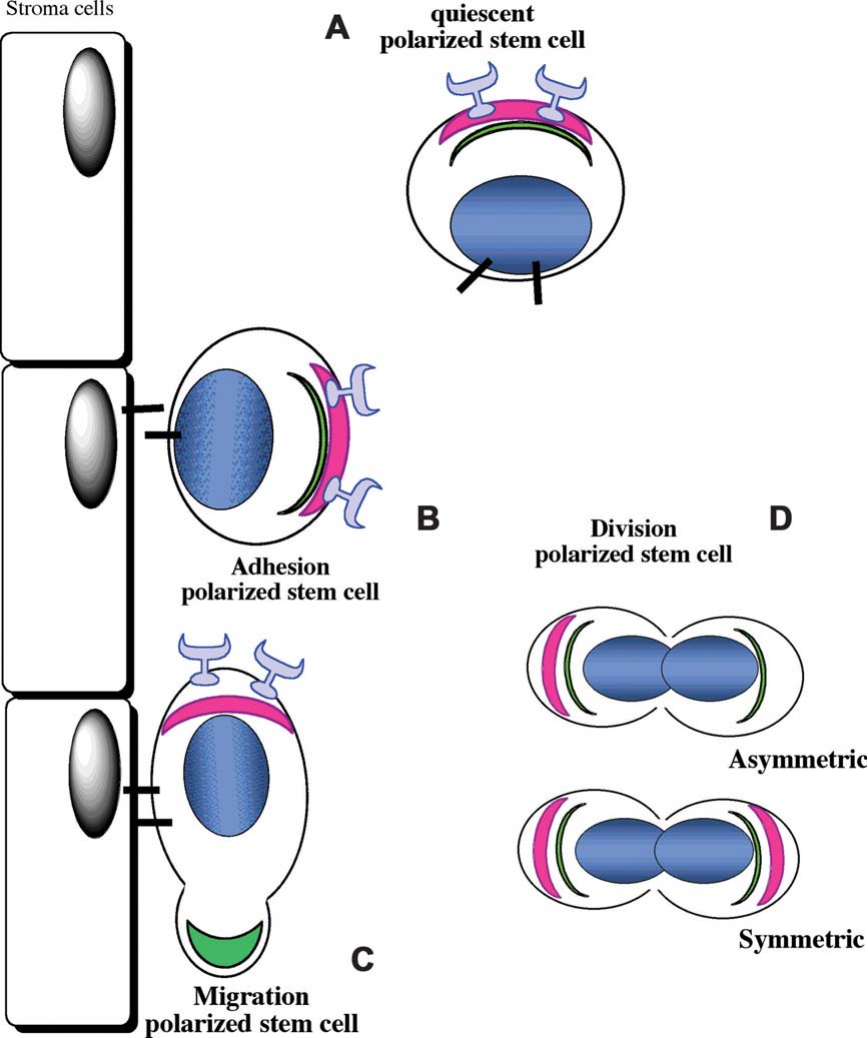
Stem cells and polarity. **(A):** Polarized quiescent stem cell. Polarity can be intrinsically established inside the cell. **(B):** An adherent polarized stem cell. Stem cell polarity can be established or maintained and reinforced upon adhesion to the niche. **(C):** Migrating polarized stem cell. **(D):** Polarized stem cells on division can distribute proteins symmetrically or asymetrically.

A defining feature of stem cells is their ability to continuously maintain a balanced number of stem cells (self-renewal) while being able to generate specialized progeny (differentiation). An additional feature of stem cells is their ability to migrate. Therefore, stem cells are unique as they are able to balance four possible fates within a single cell: quiescence, migration, proliferation, and differentiation. These fate decisions are made in the context of the supporting stroma cells they adhere to, also referred to as the niche (Fig. 1). Is there a role for polarity in stem cell fate decisions?

Stem cells that divide asymmetrically have to orient their mitotic spindle to allow for cell fate determinants to segregate asymmetrically into daughter cells. In theory, stem cells could also divide exclusively symmetrically, provided that extrinsic signals postdivision induce their postmitotic cell fate (inductive postdivision model). Growing experimental evidences demonstrate that stem cells have the ability to divide asymmetrically in vitro and in vivo, determining stem cell fate [31,32,33–35]. Even more interestingly, Wu et al. demonstrated that hematopoietic stem cells (HSCs) can undergo both symmetric and asymmetric division, and that the balance between them is not hard-wired but responsive to extrinsic and intrinsic cues [36–38].

One obvious hypothesis currently supported by experimental evidence is that polarity establishment during mitosis regulates the mode/outcome (symmetric vs. asymmetric) of stem cell divisions. For example, in *D. melanogaster*, male germ line stem cells (GLSCs) are attached to somatic hub stem cells, which constitute the stem cell niche. On division, GLSCs polarize and produce one daughter cell or gonialblast that initiates differentiation and one daughter stem cell, which remains attached to the hub [16,39–43]. Asymmetric GLSC division is controlled by the orientation of the mitotic spindle, and by the programmed anchoring of the mother centrosome to the self-renewing stem cell, while the gonialblast receives the newly synthesized centrosome [31,16,34]. A stem cell in close contact with its niche will thus orient its mitotic spindle perpendicularly to the niche surface, ensuring that only one daughter cell maintains contacts with the niche, and thus retains the ability to self-renew. Signaling molecules such as Dpp and Hh, the bone-morphogenic protein (BMP)2/4 homolog in *D. melanogaster*, released from the niche/hub cells are involved in regulating the mode of division, implying an extrinsic regulation of this polarity by the niche.

In the developing mouse brain, progenitors located in the apical surface of the ventricular zone are self-renewing and display an apical-basal polarity with a basolateral domain in contact with the basement membrane [1]. The nuclei of these neuronal precursors move basally away from the ventricular surface for DNA synthesis, and apically return to the surface for mitotic division; a process known as interkinetic migration or “to-and-fro” nuclear translocation [44,45]. In comparison with progenitors located in the subventricular zone that gradually deplete, ventricular zone progenitors contain a specialized apical membrane domain whose activity is regulated by Cdc42. In these cells, *Cdc42* deletion results in an immediate increase of basal mitosis, a gradual loss of apical membrane protein location, and increasing failure of apically directed interkinetic nuclear migration. Therefore, these Cdc42-deficient progenitors acquire the fate of the progenitors located in the subventricular zone that cannot self-renew for long time and gradually deplete [1]. It was also recently demonstrated that a planar cell polarity pathway activated by Wnt7*a* controls the number of muscle stem cells and the regenerative potential of muscle tissue [46], again most likely by regulating the mode of stem cell divisions.

**Figure 2:**
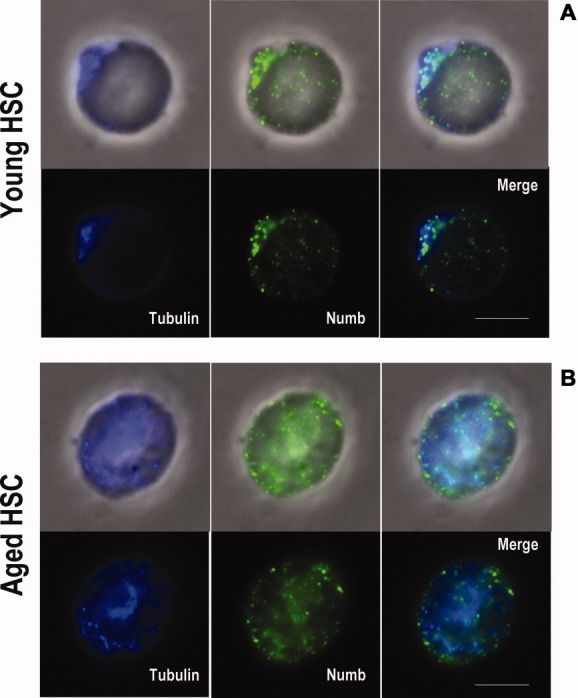
Polarized **(A)** and not polarized **(B)** HSCs. The picture is representative of tubulin (blue) and Numb (green) localization in freshly isolated mouse Lineage^−^ c-kit^+^ Sca-1^+^ CD34^−^ Flk2^−^ HSCs (long-term repopulating HSCs) from **(A)** young (2- to 4-month old) and **(B)** aged (24- to 26-month old) C57BL/6 mice. Scale bar = 5 μm. Abbreviation: HSC, hematopoietic stem cell.

On division, murine HSCs distribute Numb asymmetrically to daughter cells, a mechanism already described for asymmetric division of *D. melanogaster* neuroblasts [47]. Mammalian Numb displays a complex pattern of functions such as controlling cell fate decision, endocytosis, cell adhesion, cell migration, and ubiquitination of specific substrates and can interact with several signaling pathways (i.e., Notch, Hedgehog, p53). Alterations of Numb-dependent events and/or of Numb distribution during asymmetric cell division suggest an important role for Numb in disease and cancer progression [48] (Fig. 2).

An additional example implying a role for polarity in stem cell function comes from analysis of human hematopoietic stem/progenitor cells that can differentially localize the tetraspanins CD53, CD63, the transferrin receptor or CD71, and CD62 or l-selectin while dividing in vitro [32,35]. An asymmetric distribution of cellular components on division has also been shown by a fluorescent Notch-activity indicator system [36]. Cytokine distribution has been shown to correlate with cell fate determination on division [49]; however, it is unclear whether cytokines are actually instructive in this process. Collectively, these and other published data support that both modes of cell division (asymmetric/polar and symmetric/nonpolar) are used by HSCs, with both intrinsic as well as extrinsic signals determining polarity on division.

Recently, a stem cell stroma synapse-like structure has been postulated in analogy to the well-characterized immune cell synapse [50–52] that describes the contact plane between T-cells and antigen-presenting cells [53–55]. Recent data demonstrating polarity in nondividing HSCs interacting with niche cells support a role for polarity in the stem cell synapse, like the reported T-cell-polarity on interaction with antigen-presenting cells [56,57]. Adult stem cells residing in their niche are mostly in a quiescent cell cycle state. Although cell cycle quiescence has so far not been frequently associated with cellular polarity, recent results analyzing mice deficient for Cdc42 in HSCs suggest that polarity established by Cdc42 might be necessary for both adhesion of HSCs to the niche as well as their quiescence, as these mice show an increase in the number and the frequency of phenotypic short-term HSCs and a loss of long-term HSCs [58,59]. Therefore, albeit supported by only few experimental results so far, polarity might be necessary in maintaining HSC quiescence by functioning in the formation of the stem cell-niche synapse, and polarity alterations might importantly impair stem cell quiescence or function. Such a polarity-based synapse model though leaves the question open whether adhesion to the niche induces polarity in stem cells (extrinsic regulation of polarity) or whether stem cells present an intrinsic polarity axis, in which a polar interaction with the niche might only be secondary to this intrinsically established polarity [16,50–51].

Although polarity in migration has been extensively studied in differentiated progeny of stem cells-like neutrophils [5,60,61], the role of polarity in stem cell migration has not been investigated in great detail. Obviously, more research in this area is necessary, although there is evidence that stem cell migration and migration-associated polarity are also regulated by small RhoGTPases [62].

## STEM CELL POLARITY IN CANCER AND AGING

Whether there is a causal relationship between altered stem cell polarity and cancer initiation and propagation is still a matter of debate. The laboratory of P. G. Pellicci has recently demonstrated that targeted mutation of the tumor suppressor *p53* in mammary stem cells increases the frequency of stem cell symmetric division thus increasing susceptibility to tumor development in the mammary gland [63]. The data from this model imply that p53 regulates polarity on cell division in mammary stem cells and suggest that loss of p53 favors symmetric division, contributing to tumor initiation [63]. Stem cell polarity disruption could also be linked to colorectal cancer. Several studies have shown that mutations in the tumor suppressor gene adenomatosis polyposis coli (APC) occur primarily in the basal crypt colonic stem cells, which are presumed tumor initiating colorectal cancer stem cells. APC loss is altering Wnt signal transduction and is ultimately influencing both cell proliferation and polarity, leading to an accumulation of symmetric dividing dysplastic cells emanating from the base of the crypt toward the surface [64].

Evidence linking polarity and aging was initially described in yeast. For example, Shcheprova et al. showed that during budding, aging factors (such as carbonylated proteins and extrachromosomal ribosomal DNA circles or endoplasmatic reticulum-resident calcium binding protein (ERC)) are asymmetrically segregated in the mother cell, and the presence of a barrier between mother and daughter cell ensures the confinement of these factors, demonstrating a mechanism for asymmetric protein segregation regulating aging during yeast budding. When the mother cell ages, the barrier breaks down and aging factors can pass to the daughter cell [65,66]. Additional experiments in bacteria and yeasts in summary suggest that cell polarization may have evolved to restrict senescence to one daughter cell during division by enabling the differential segregation of damaged or old material [67]. Therefore, aging seems to be intricately connected to polarity [68].

Stem cells undergo a time-dependent functional decline, at least in those tissues in which the cellular turnover is high (i.e., germ line, blood, gut epithelium, and epidermis) [69–74]. Recent data indicate a correlation between altered stem cell polarity and stem cell aging. For example, in *Drosophila* species, aged GLSCs exhibit misoriented centrosomes and thus altered polarity relative to their niche cells. This correlates with a reduced self-renewal activity of aged GLSCs most likely due to the alteration in the mode of stem cell division [75]. Whether similarly to yeast, polarity allows stem cells to differentially retain or transmit aging factors to daughter cells and whether this generational asymmetry might then result in age-dependent differences in the biochemical composition of “old” and “new” stem cells, even under symmetric divisions is still an open question.

In the hematopoietic system, aging alters/reduces stem cell function with regard to mobilization, homing, engrafting, and lineage choice [76–80]. Aged HSCs show reduced polarity with respect to the distribution of the established polarity markers tubulin and numb (Fig. 2) [57]. These data correlate with reduced self-renewal and altered differentiation of aged HSCs. In analogy with what is already described in yeast, the reduced polarity in aged HSCs might be linked to elevated activity of Cdc42 [81]. Interestingly, it has also been shown that Cdc42 expression increases in human lymphoblastoid cell lines with the age of the donor, and more importantly that after adjusting for the age, higher expression of Cdc42 in these human lymphoblastoid cell lines is associated with increased donor mortality [82]. This observation correlates with the finding that elevated activation of Cdc42 is associated with aging in multiple tissues in mice, and that mice with constitutively active Cdc42 manifest signs of a premature aging-like syndrome [77,83].

However, whether Cdc42-regulated polarity is also involved in aging in *Drosophila* GLSCs has not been reported. In summary, the data published so far imply an important and causative role of cell polarity in multiple aspects of stem cells in cancer and aging.

## CONCLUSION

Polarity is essential for the survival of almost all organisms. Determining the role of cellular polarity for stem cell function in particular is still a very new field of research. Summarizing recent findings, the data support a concept in which alterations in polarity are important in various aspects of stem cell behavior from *Drosophila* species to mice. This implies that proper establishment of polarity is a universal attribute for a successful life of almost all types of cells and organisms [68]. Consequently, procedures/drugs that are able to alter cell polarity in stem cells in either disease or aging might be regarded as novel approaches to revert multiple aspects of somatic stem cell disease and aging.

## Disclosure of Potential Conflicts of Interest

The authors indicate no potential conflicts of interest.
